# The potential effectiveness of far-UVC (222 nm) in preventing infections in long-term care facilities: a six-month nonrandomized controlled phase II trial

**DOI:** 10.1017/ash.2026.10425

**Published:** 2026-06-03

**Authors:** Mette Assenholm Kristensen, Emilie Hage Mogensen, Stine Yde Nielsen, Christian Kanstrup Holm

**Affiliations:** 1 Department of Microbiology, Lillebaelt Hospital - University Hospital of Southern Denmark: Sygehus Lillebalt, Vejle, Denmark; 2 UV Medico A/S, Denmark; 3 Department of Clinical Microbiology, Lillebaelt Hospital - University Hospital of Southern Denmark: Sygehus Lillebalt, Vejle, Denmark; 4 Aarhus University: Aarhus Universitet, Denmark

## Abstract

**Objectives:**

The objectives of this study were to assess the potential effectiveness of far-UVC (222 nm) in reducing hospital-treated infections, antibiotic prescriptions, and mortality in long-term care facilities (LTCFs), as well as to evaluate the rationale for a future randomized controlled trial (RCT).

**Design:**

A six-month, nonrandomized, controlled, three-arm Phase II trial was conducted in LTCFs in Denmark. Facilities were allocated in a 10:1:1 ratio to usual care, far-UVC in common rooms, and far-UVC in common and residents’ rooms. The trial was conducted between November 2024 and April 2025. The primary outcome was LTCF-acquired infections treated in hospitals.

**Results:**

The study included 12 LTCFs with a total of 635 residents. In the usual care group (n = 470), 600 hospital-treated infections were identified with a incidence rate (IR) of 86.2 per 10,000 resident-days. In the common rooms group (n = 76), 12 infections were identified with a IR of 10.4 per 10,000 resident-days and an adjusted incidence rate ratio (aIRR) of 0.12 (95% CI: 0.04–0.34). In the common and residents’ rooms group (n = 89), 78 infections were identified with a IR of 59.1 per 10,000 resident-days and an aIRR of 0.69 (95% CI: 0.23–2.07). The aIRR for total antibiotic prescriptions was 0.52 (95% CI: 0.29–0.96) in the group with lamps in common rooms and 0.83 (95% CI: 0.50–1.37) in the group with lamps in common and residents’ rooms.

**Conclusions:**

Far-UVC potentially reduces hospital-treated infections and antibiotic prescriptions in LTCFs, but these results should be considered preliminary until confirmed by an RCT.

## Introduction

Elderly residents in long-term care facilities (LTCFs) are particularly vulnerable to infections due to age-related changes, comorbidities, and frequent exposure.^1^ Infection prevalence among LTCF residents ranges from 2.3% to 5.1%, with urinary tract infections and pneumonia being the most common.^2^ If not fatal, these infections often require hospitalization, which is distressing for residents and their families as well as costly for society. Hospital undercapacity is a persistent concern, especially during seasonal peaks and outbreaks. As the global population ages, the number of individuals aged over 80 years is projected to triple from 143 million in 2019 to 426 million in 2,050,^3^ increasing the demand for long-term care.^4^ The COVID-19 pandemic highlighted the vulnerability of LTCF residents, who account for up to 50% of reported deaths globally, although mortality rates varied across countries.^5^

The pandemic also broadened understanding of airborne transmission, with accumulating evidence indicating that most respiratory viruses spread primarily via small and large aerosols,^6^ collectively termed “infectious respiratory particles.”^7^ Preventive measures such as masking, physical distancing, and air disinfection are therefore key components of strategies to reduce airborne transmission. However, rigorous infection control measures during the pandemic led to substantial social isolation among LTCF residents,^8^ highlighting the need for new strategies to reduce transmission. Effective nonpharmacological interventions may also help keep society more open while vaccines and treatments are developed, especially for pathogens with efficient transmission from asymptomatic or mildly symptomatic individuals, such as SARS-CoV2-2.^9^ Most pathogens, including multi-drug resistant organisms, can survive on surfaces for days or weeks, and contribute to the transmission of pathogens.^10^ The rise of *Clostridoides (C.) difficile,* newly emerging pathogens such as *Candida auris* and multidrug-resistant Gram-negative bacteria producing carbapenemases^11^ has made it increasingly attractive to prevent environmental contamination. Thus, alternatives to current cleaning practices and chemical disinfection, such as ultraviolet light, novel disinfectants, and probiotic-based cleaning,^12^ are emerging.

Far-UVC (200–235 nm) has recently gained attention for its potential to reduce disease transmission by inactivating micro-organisms in the air and on surfaces,^13–15^ including influenza viruses,^16^ SARS-CoV-2^17^ and multi-drug resistant organisms.^18^ When applied within regulatory exposure limits, far-UVC has been shown to be safe for skin and eyes.^19–21^ To date, no studies have evaluated the impact on infection rates, antibiotic use, or mortality among elderly LTCF residents. We therefore conducted a phase 2 trial to assess the potential effectiveness of far-UVC in reducing hospital-treated infections, antibiotic prescriptions, and mortality in LTCFs, and to evaluate the rationale for a future randomized controlled trial (RCT).

## Methods

### Trial design

A six-month controlled, three-arm, phase II trial was conducted in public LTCFs in Vejle, Denmark, from 1 November 2024 to 30 April 2025, as described in the trial protocol.^22^ The trial used a parallel, non-randomized, open-label design with blinded analysis. LTCFs were allocated in a 10:1:1 ratio to usual care versus one of two intervention groups receiving far-UVC installation in either common rooms only or in both common rooms and residents’ rooms. The trial adhered to the CONSORT 2025 guideline and relevant extension for multi-arm and pilot or feasibility trials^23–25^ where applicable. The Ethics Committee of the Regional Committees on Health Research Ethics for Southern Denmark approved the study protocol in June 2024 (registration S-20242000-106). This trial was registered at ClinicalTrials.gov (NCT07511881) in April 2026 after enrollment.

### Changes to trial protocol

We expanded ozone (O_3_) monitoring due to concerns about potential harmful O_3_ generation from far-UVC lamps. Preliminary measurements showed concentrations below the detection limit of our monitoring instrument (EcoZone Monitor, model EZ-1X, Scanion, Denmark). However, existing evidence is mostly limited to small, unoccupied settings.^26^ Therefore, we conducted a more comprehensive O_3_ assessment in occupied LTCFs with far-UVC, the results of which will be reported separately.

Additional protocol changes included omitting patient comorbidity variables and the outcome measuring cause-specific death due to few events (n < 5), as well as excluding questionnaire-based harm data due to low data quality. We added infections of unknown focus to the main analysis and conducted a post hoc pooled intervention analysis, resulting in a two-arm comparison.

### Trial setting

The trial was conducted in public LTCFs^27^ in Vejle Municipality, Denmark.^22^ In Denmark, elderly individuals typically move into LTC facilities only when their needs cannot be met through home care. Therefore, frailty and level of care are high in LTCFs. All LTCF residents had private rooms with a bathroom, kitchenette, and access to shared indoor and outdoor common areas. Infection control guidelines in Danish LTCFs follow a multimodal prevention strategy, including strengthening host defenses in residents, preventing pathogen transmission through standard precautions, and diagnosing and treating infections. Isolation precautions are not routinely practised.^28^

All Danish citizens are assigned a unique 10-digit Central Personal Register number, enabling linkage of individual-level data across public registers.^29,30^

### Eligibility criteria for sites and participants

We excluded LTCFs if they had non-standardized building types and layouts, shared common areas with kindergarten, or were specialized dementia facilities. All residents with permanent stays were eligible for inclusion, and individuals moving into participating LTCFs during the intervention period were enrolled.

### Intervention

The intervention consisted of installing far-UVC lamps (UV222, UV Medico A/S, Denmark) emitting filtered UV light at 222 nm.^22^ We created three-dimensional room models, incorporating lamp placement and typical occupancy patterns, to optimize mounting angle, duty cycles, and UV light distribution while complying with exposure regulations (23 mJ/cm^2^ per day).^20^ Modeling was performed using DIALux evo version 9.2 (DIAL GmbG, Germany).

In common rooms, ceiling-mounted lamps were installed with a 30° tilt and operated on a 2:1 on/off duty cycle. In residents’ rooms, lamps were mounted on wheeled stands, placed in a corner of the bedroom, and oriented away from the bed.

In the LTCF equipped with far-UVC in common rooms only, five lamps were installed in each of six common rooms (78 m^2^) and operated from 7:00 am to 8:00 pm (average fluence of 1.27 µW/cm^2^). In the LTCF equipped with far-UVC in both common and residents’ rooms, nine lamps were installed in a large common room (226 m^2^) and operated from 7:30 am to 6:15 pm (average fluence of 1.05 µW/cm^2^). Three smaller common rooms (66 m^2^ each) contained five lamps operating from 7:00 am to 8:00 pm (average fluence rate of 1.26 µW/cm^2^). Each of 72 resident’s room (15 m^2^) contained one lamp operating from 7:00 am to 8:00 pm (average fluence of 0.93 µW/cm^2^). The Far-UVC lamps were turned off during night-time hours in residents’ rooms to minimize disturbance, as the characteristic purple glow of krypton chloride light sources may otherwise interfere with sleep.

### Outcomes

Outcome was the number of hospital contacts (hospital stay ≥ 12 h and acute hospital outpatient visits) due to selected LTCF-acquired infections, including upper and lower respiratory tract infections, urinary tract infections, bloodstream infections, and infections of unknown focus. We identified the LTCF-acquired infections using: (1) hospital discharge diagnosis based on the International Classification of Diseases, 10th Revision (ICD-10) codes, and/or: (2) the prescription of antibiotics or antiviral agents covering the included infection types within 48 hours of hospital admission, classified using the Anatomical Therapeutic Chemical codes. Treatments lasting less than 24 hours were excluded. Either criterion was sufficient for classification.

To minimize misclassification, infections had to occur more than seven days after enrollment and more than 14 days after prior hospitalization.

A new infection was included if it occurred at least 30 days after a previous infection of the same anatomical site of infection or at any time within 30 days of a different type. Residents with frequent (> 6) or prolonged (> 2 mo) hospitalizations during the trial were excluded due to uncertainty regarding infection acquisition.

Another outcome was systemic antibiotics prescribed by general practitioners for non-hospitalized residents, categorized as respiratory (excluding antivirals), urinary tract, skin, or other. All-cause mortality was also assessed. These outcomes were included only if they occurred more than seven days after enrollment.

Residents remained part of the trial until death, relocation from the LTCF, or the end of follow-up (30 April 2025). Participant timelines were measured in resident days.

### Statistical methods

We conducted an intention-to-treat analysis. Incidence rates (IRs) were calculated using resident days as the denominator and the number of events as the numerator. We then estimated incidence rate ratios (IRRs), defined as the ratio of these IRs between groups, using Poisson regression with a robust standard error. In a multivariable regression, we estimated an adjusted incidence rate ratio. We adjusted for age only. We used prespecified subgroup analysis to examine whether the estimated effectiveness differed by a Charles Comorbidity Index (CCI) score ≥2^31^ and age >85 years. We did not adjust for age in the subgroup analysis, age >85 years, to avoid including the same predictor variable twice. Moreover, we performed a post hoc analysis in which the two intervention groups were pooled into one single intervention group and analyzed using the same analytical approach. Counts <5 and the accompanying analyses are not reported in accordance with guidelines for handling personally identifiable data. Two-sided *P*-values of <0.05 were considered statistically significant. All statistical analyses were conducted using Stata version 17 (StataCorp, College Station, Texas, USA) by an epidemiologist (non-blinded) and M.A.K (blinded).

## Results

### Baseline characteristics

**Figure 1.**
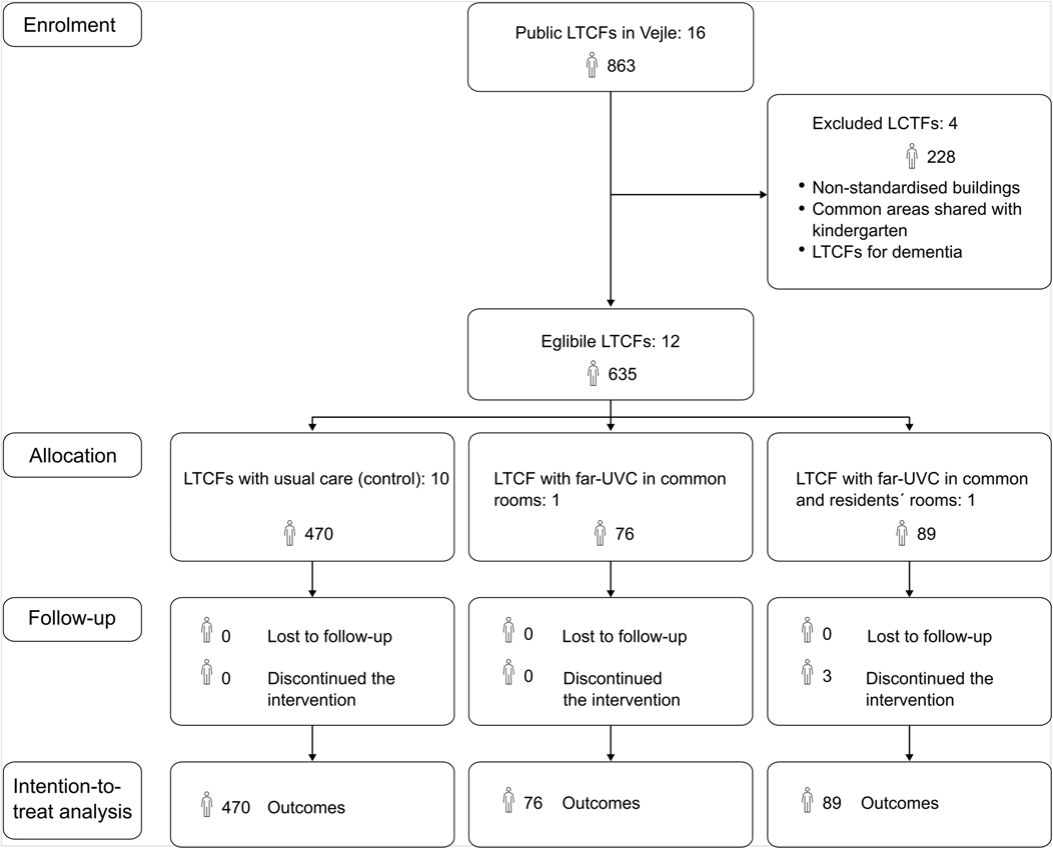
Figure 1 long description. Flow diagram of the trial, showing enrolled long-term care facilities, number of participating residents, intervention allocation, and residents included in the analysis.

Of 16 LTCFs assessed, 12 met the inclusion criteria (Figure 1), comprising 635 residents. Seventy-six residents were allocated to the intervention group with far-UVC in common rooms, and 89 to the intervention group with far-UVC in both common and residents’ rooms. In the latter group, three individuals did not receive far-UVC in their private rooms due to space constraints or discomfort. The usual care group comprised 470 residents.

The baseline characteristics of residents are presented in Table 1. Overall, the resident characteristics of the groups were very similar. However, residents in the common rooms group were younger than those in the other groups (test not shown).

**Table 1.** Baseline demographic and clinical characteristics of participants by group. Data are numbers (%) unless stated otherwise Table 1 long description.

Characteristics N = 635	Far-UVC in common rooms (n = 76)	Far-UVC in common and residents’ rooms (n = 89)	Usual care (n = 470)
Age			
< 75 yr	23 (30.3)	8 (9.0)	86 (18.3)
75–85 yr	27 (35.5)	37 (41.6)	167 (35.5)
> 85 yr	26 (34.2)	44 (49.4)	217 (46.2)
Mean (SD) yr	79.2 (11.2)	84.9 (8.2)	82.9 (9.7)
Median yr	83.0	85.0	85.0
Sex			
Male	32 (42.1)	27 (30)	182 (38.7)
Female	44 (57.9)	62 (69.7)	288 (61.3)
Charlson comorbidity index score			
≤ 1	47 (61.8)	58 (65.2)	291 (61.9)
≥ 2	29 (38.2)	31 (34.8)	179 (38.1)
Vaccine status			
Pneumococcal	56 (73.7)	63 (70.8)	348 (74.0)
Influenza	69 (90.8)	83 (93.3)	432 (91.9)
COVID-19	72 (94.7)	85 (95.5)	455 (96.8)

The baseline characteristics of LTCFs are presented in Table 2. All included LTCFs were constructed between 1975 and 2006. Air purifiers were frequently used in smoking areas or for odor control, except in the LTCF withfar-UVCin common rooms. Residents’ rooms were cleaned every two weeks across all facilities. Cleaning frequency in common rooms varied: daily in the LTCF with far-UVC in common rooms, weekly in the other intervention group, and several times per week (40%), weekly (50%), or daily (10%) in the usual care group. During autumn and winter, residents in all groups predominantly stayed indoors. Staff-to-resident ratios and occupant density in all groups were very similar.

**Table 2.** Baseline characteristics of long-term care facilities by group. Data are numbers (%) unless stated otherwise Table 2 long description.

Characteristics	Far-UVC in common rooms (n = 1)	Far-UVC in common and residents’ rooms (n = 1)	Usual care (n = 10)
Measures related to building			
Ventilation			
Natural ventilation	0 (0)	0 (0)	3 (30)
Mechanical ventilation	0 (0)	0 (0)	5 (50)
Waste heat recovery unit	1 (100)	1 (100)	2 (20)
Air filtration			
No filter	0 (0)	0 (0)	5 (50)
Coarse and fine filter	1 (100)	1 (100)	5 (50)
High-efficiency particulate air filter	0 (0)	0 (0)	0 (0)
Air purifier	0 (0)	1 (100)	9 (90)
Last renovation, year	0	1, 2008	5, 2002 to 2024
Building age, year	2006	1980	1975 to 2005
Measures related to organization			
Permanent staff-to-resident, ratio^a^	68/66, 1.0	75/72, 1.0	480/404, 1.2
Occupant density, ratio^b^	66/4896, 0.01	72/5472, 0.01	404/38596, 0.01
Cleaning frequency, residents’ home			
Weekly	0 (0)	0 (0)	1 (10)
Every second week	1 (100)	1 (100)	90 (90)
Cleaning frequency, common areas			
Every day	1 (100)	0 (0)	1 (10)
Several times a week	0 (0)	0 (0)	4 (40)
Weekly	0 (0)	1 (100)	5 (50)
Whereabouts of the residents			
Outdoor, March to September			
< 2 h	0 (0)	0 (0)	2 (20)
2–6 h	1 (100)	1 (100)	8 (80)
7–10 h	0 (0)	0 (0)	0 (0)
11–16 h	0 (0)	0 (0)	0 (0)
Outdoor, October to February			
< 2 h	1 (100)	1 (100)	9 (90)
2–6 h	0 (0)	0 (0)	1 (10)
7–10 h	0 (0)	0 (0)	0 (0)
11–16 h	0 (0)	0 (0)	0 (0)

a Permanent staff per resident.

b Residents per m^2^.

### Potential effectiveness of far-UVC on infections

Table 3 presents the outcome examining the potential effectiveness of far-UVC in reducing infections requiring hospitalization. During the trial, 690 infections occurred during 94,325 resident days. The aIRR for total infections was 0.12 (95% CI: 0.04–0.34) in the group with far-UVC in common rooms and 0.69 (95% CI: 0.23–2.07) in the group with far-UVC in common and residents’ rooms. Table 3 presents the results by infection type.

**Table 3.** Results of hospital-treated infections. Events and incidence rates (IRs) per 10,000 resident days, and unadjusted and adjusted incidence ratios (IRRs) between the two intervention groups and the usual care group Table 3 long description.

N = 635	Events, IR			Unadjusted IRR (95%CI)		Adjusted^b^ IRR (95%CI)	*P*-Value	Adjusted^b^ IRR (95%CI)	*P*-Value
Outcome	Far-UVC in common rooms 11,536 resident days (n = 76)	Far-UVC in common and residents’ rooms 13,204 resident days (n = 89)	Usual care 69,585 resident days (n = 470)	Far-UVC in common rooms	Far-UVC in common and residents’ rooms	Far-UVC in common rooms	–	Far-UVC in common and residents’ rooms	–
Infections, all^a^	12, 10.4	78, 59.1	600, 86.2	0.12 (0.04–0.36)	0.69 (0.23–2.06)	0.12 (0.04–0.34)	.000	0.69 (0.23–2.07)	.505
Urinary tract infections	< 5	23, 17.4	172, 24.7	’.’	0.70 (0.23–2.16)	’.’	’.’	0.71 (0.23–2.16)	.545
Respiratory tract infections	6, 5.2	27, 20.4	201, 28.9	0.18 (0.06–0.53)	0.71 (0.25–2.02)	0.18 (0.06–0.51)	.001	0.70 (0.25–2.00)	.507
Bloodstream infections	< 5	15, 11.4	144, 20.7	’.’	0.55 (0.32–0.94)	’.’	’.’	0.56 (0.15–2.13)	.398
Unknown infection focus	< 5	13, 9.8	83, 11.9	’.’	0.83 (0.18–3.72)	’.’	’.’	0.81 (0.18–3.77)	.796

a Urinary tract infections, respiratory tract infections, bloodstream infections, including infections of unknown focus (ICD-10 codes A49, B99), and antibiotics covering unknown infection focus (J01CA01 and J01GB03 or J01CR05 or J01GB03 and J01DC02).

b Adjusted for age.

In the subgroup analysis (Table 4), the aIRR for total infections among residents with CCI ≥2 was 0.54 (95% CI: 0.09–3.14) in the group with far-UVC in common and residents’ rooms. For residents aged over 85 years, the IRR for total infections was omitted due to counts >5. Results by infection type are shown in Table 3.

**Table 4.** Results of main and subgroup analyses of hospital-treated infections and antibiotic prescriptions in long-term care facilities among residents aged >85 years with CCI ≥2 Table 4 long description.

	Events, IR			Unadjusted IRR (95%CI)		Adjusted^d^ IRR (95%CI)	*P*-Value	Adjusted^d^ IRR (95%CI)	*P*-Value
Outcome	Far-UVC in common rooms	Far-UVC in common and residents’ rooms	Usual care	Far-UVC in common rooms	Far-UVC in common and residents’ rooms	Far-UVC in common rooms	–	Far-UVC in common and residents’ rooms	–
Infections, all^a^ N = 635 (main)	12, 10.4	78, 59.1	600, 86.2	0.0 (0.23–2.06)	0.69 (0.23–2.06)	0.12 (0.04–0.34)	.000	0.69 (0.23–2.07)	.505
CCI^b^ ≥2 n = 239 (subgroup)	< 5 4,218 resident days	39, 87.6 4,453 resident days	475, 175.7 27,035 resident days	’.’	0.50 (0.09–2.78)	’.’	’.’	0.54 (0.09–3.14)	.490
Years >85 n = 287 (subgroup)	< 5 4,089 resident days	< 5 6,480 resident days	198, 62.8 31,547 resident days	’.’	’.’	’.’	’.’	’.’	’.’
Antibiotic prescriptions, all^c^ N = 635 (main)	29, 25.1	52, 39.4	334, 48.0	0.52 (0.29–0.95)	0.82 (0.52–0.16)	0.52 (0.29–0.96)	.036	0.83 (0.50–1.37)	.461
CCI^b^ ≥2 n = 239 (subgroup)	4,218 resident days 5, 11.9	4,453 resident days 17, 38.2	27,035 resident days 123, 37.83	0.26 (0.09–0.73)	0.84 (0.37–1.89)	0.26 (0.09–0.73)	.011	0.78 (0.34–1.76)	.545
Years >85 n = 287 (subgroup)	4,089 resident days 9, 22.0	6,480 resident days 27, 41.7	31,547 resident days 171, 54.2	0.41 (0.18–0.93)	0.77 (0.40–1.47)	’.’	’.’	’.’	’.’

a Urinary tract infections, respiratory tract infections, bloodstream infections, including infections of unknown focus (ICD-10 codes A49, B99), and antibiotics covering infections of unknown focus (J01CA01 and J01GB03 or J01CR05 or J01GB03 and J01DC02).

b Charlson Comorbidity Index.

c Urinary tract infections, respiratory tract infections, skin infections, and other infections.

d Adjusted for age.

### Potential effectiveness of far-UVC on antibiotic prescriptions and mortality

The aIRR for total antibiotic prescriptions was 0.52 (95% CI: 0.29–0.96) in the group with far-UVC in common rooms and 0.83 (95% CI: 0.50–1.37) in the group with far-UVC in common and residents’ rooms. The aIRRs for mortality were 0.84 (95% CI: 0.43–1.66) and 0.83 (95% CI: 0.46–1.50), respectively.

In the subgroup analysis (Table 4), the aIRR for total antibiotic prescriptions among residents with CCI ≥2 was 0.26 (95% CI: 0.09–0.73) in the group with far-UVC in common rooms and 0.78 (95% CI: 0.34–1.76) in the group with far-UVC in common and residents’ rooms. Among residents aged over 85 years, the IRRs were 0.41 (95% CI: 0.18–0.93) and 0.77 (95% CI: 0.40–1.47), respectively. Table 5 presents the results of antibiotic prescription by infection type.

**Table 5.** Results of antibiotic prescriptions in long-term care facilities and mortality. Events and incidence rates (IRs) per 10,000 resident days, unadjusted and adjusted incidence ratios (IRRs) between the two intervention groups and the usual care group Table 5 long description.

N = 635	Events, IR			Unadjusted IRR (95%CI)		Adjusted^b^ IRR (95%CI)	*P*-Value	Adjusted^b^ IRR (95%CI)	*P*-Value
Outcome	Far-UVC in common rooms 11,536 resident days (n = 76)	Far-UVC in common and residents’ rooms 13,204 resident days (n = 89)	Usual care 69,585 resident days (n = 470)	Far-UVC in common rooms	Far-UVC in common and residents’ rooms	Far-UVC in common rooms	–	Far-UVC in common and residents’ rooms	–
Antibiotic prescriptions, all^a^	29, 25.1	52, 39.4	334, 48.0	0.52 (0.29–0.95)	0.82 (0.52–0.16)	0.52 (0.29–0.96)	.036	0.83 (0.50–1.37)	.461
Antibiotic prescriptions, urinary tract	8, 6.9	29, 22.0	157, 19.2	0.54 (0.30–1.00)	1.19 (0.61–2.30)	0.56 (0.31–1.04)	.067	1.15 (0.59–2.22)	.684
Antibiotic prescriptions, respiratory tract	19, 16.5	18, 13.6	129, 16.5	1.57 (0.81–3.05)	0.90 (0.51–1.57)	1.48 (0.76–2.88)	.996	0.99 (0.56–1.68)	.996
Antibiotic prescriptions, skin	< 5	5, 3.8	42, 6.0	’.’	0.77 (0.2–2.54)	’.’	’.’	0.96 (0.06–3.54)	.950
Antibiotic prescriptions, other	0, 0.0	0, 0.0	6, 0.86	’.’	’.’	’.’	’.’	’.’	’.’
Mortality	10, 8.7	13, 9.85	78, 11.2	0.77 (0.40–1.50)	0.89 (0.49–1.59)	0.84 (0.43–1.66)	.624	0.83 (0.46–1.5)	.543

a Urinary tract infections, respiratory tract infections, skin infections, and other infections.

b Adjusted for age.

In a post hoc analysis pooling the intervention groups, the aIRR for total infections was 0.40 (95% CI: 0.15–1.1) compared with usual care, and the aIRR for total antibiotic prescriptions was 0.68 (95% CI: 0.53–0.87). The results for specific infection types requiring hospitalization and antibiotic prescriptions by infection type are presented in Supplementary Table 1.

During monitoring for protocol adherence, we identified a small number of far-UVC lamps that were unplugged.

## Discussion

In this phase II trial, we investigated the potential effectiveness of far-UVC as a non-pharmacological intervention to reduce the number of hospital-treated infections, antibiotic prescriptions and mortality in LTCFs.

We found that the use of far-UVC was associated with hospital-treated infections, antibiotic prescriptions, and mortality, warranting further investigation in a large-scale randomized phase III trial. Across all trial groups, 690 infections resulted in hospital contact for 94,235 resident-days. Facilities with far-UVC in common rooms showed a strong association in hospital-treated infections, whereas facilities with far-UVC in common and residents’ rooms showed a moderate association. In both intervention groups, stronger associations were observed among residents aged over 85 years, and those with higher CCI scores, although parts of the analysis were omitted due to counts >5. Antibiotic prescriptions showed a weak association with far-UVC, but with a similar pattern of stronger associations in these subgroups. There was a weak association between far-UVC and mortality. No dose–response relationship was identified. Instead, a stronger association was found in the group with far-UVC in common rooms only, compared to far-UVC in both common rooms and residents’ rooms.

Our findings build on experimental evidence that far-UVC inactivates micro-organisms in the air^32,33^ and on surfaces,^34^ suggesting potential for reduced transmission in real-world care settings. In a study of airborne bacteria, fluence rates of 0.1–2.7 µW/cm^2^ corresponded to approximately 35–184 equivalent air changes per hour,^32^ placing the fluence used in our study (0.9–1.2 µW/cm^2^) within this range. Consistent with this, another study in an occupied space reported efficient viral inactivation at a mean fluence of 0.82 µW/cm,^2^ corresponding to 1,480 equivalent air changes per hour.^33^

In our study, far-UVC was associated with respiratory infections requiring hospital contact in both the group with far-UVC in common rooms (IRR 0.18) and in the group with far-UVC in common and residents’ rooms (IRR 0.70). By comparison, an RCT of conventional germicidal UV light installed in common rooms reported only a modest reduction in acute respiratory infections (IRR 0.91).^35^

Evidence from the broader literature is less consistent. A systematic review evaluating air treatment technologies, including conventional UV light, did not find clear evidence of reduced respiratory infections.^36^ The included trials used conventional UV in private homes, a hospital ward, and an office building. The authors noted, however, that air treatment technologies may be more effective in LTCFs, where residents spend prolonged periods indoors and have increased vulnerability due to frailty and comorbidity.^36^ The stronger association observed in our study may therefore reflect the LTCF setting, advances in UV technology such as the use of far-UVC for whole-room disinfection rather than upper-room irradiation with conventional UV, or a combination of these factors. Nonetheless, the limited internal validity and statistical uncertainty inherent to a phase II trial must be considered. To our knowledge, only one ongoing RCT is currently evaluating the effectiveness of far-UVC on infection rates in LTCFs, focusing on winter respiratory infections and harms.^37^

### Limitations

Our trial had several important methodological limitations. The first and most crucial limitation is the risk of type I error and risk of bias due to the non-randomized cluster design. This is particularly important because we only estimated age-adjusted IRR, despite several important practice differences in air handling and cleaning frequency between the intervention and the usual care group. Moreover, residual confounding from other unmeasured factors may have occurred. Second, an 88% risk in the group treated with the lowest intensity of far-UVC, combined with a smaller effect in the more intensively treated group, suggests the presence of unmeasured bias and/or statistical uncertainty. Third, as a phase II trial, it was underpowered, increasing the risk of type II error. For example, the data protection–driven limitation (suppression of counts with n < 5) substantially limited our ability to assess baseline differences in the study population and differences in study outcomes. Fourth, we applied stringent criteria to distinguish LTCF-acquired infections from hospital-acquired infections. However, antibiotic prescription at the LTCFs and mortality were not assessed with the same level of accuracy. This increases the risk of inadvertent inclusion of hospital-acquired infections, which may have led to an underestimation of the association between far-UVC and antibiotic prescription at the LTCFs and mortality. Another information bias is that hospitalization due to infection identified in health registries does not distinguish between infections arising from endogenous flora and exogenous acquisition, of which only the latter are potentially preventable by UV disinfection. This limitation may lead to an overestimated association, particularly for infections that are typically endogenous, such as urinary tract infections. Moreover, antimicrobial prescribing is a well-documented occurrence in LTCFs, which also might have overestimated the association. Finally, our outcome definition did not include antiviral agents. As antiviral drugs are prescribed exclusively in hospitals and not by LTCF physicians, infections caused by, for example SARS-CoV-2 and influenza are likely underestimated for cases not requiring hospital admission/contact. Fifth, the absence of point-of-care microbiological testing performed at the LTCF testing, such as nasopharyngeal swabs for respiratory viruses, may have led to an underestimation of mild viral infections that did not require hospital care unless treated with antibiotics at the LTCFs. Both respiratory virus and bacteriological test results were available in the national microbiological database, but we did not have access to retrieve these data. Consequently, information on the causative pathogens of the infections was unavailable. Finally, we did not use trained assessors to examine each resident for illness but relied on diagnosis codes and prescribed antimicrobial agents from validated registers. This may have introduced information bias through misclassification of the resident’s illness. Due to the above-mentioned limitations, the results of far-UVC must be interpreted as preliminary until confirmed in RCTs.

In conclusion, far-UVC potentially reduces hospital-treated infections, particularly among residents with higher CCI scores. Moreover, far-UVC potentially reduces the number of antibiotic prescriptions in LTCTs. We found a minor potential reduction in mortality. The results support the rationale for a future RCT. Due to limitations, the results should be considered preliminary until confirmed by an RCT.

## Supporting information

10.1017/ash.2026.10425.sm001 Kristensen et al. supplementary material Kristensen et al. supplementary material

## Supplementary material

The supplementary material for this article can be found at https://doi.org/10.1017/ash.2026.10425.

## Figure 1. Long description

The flowchart illustrates the stages of a trial involving long-term care facilities (LTCFs). It begins with the enrollment of 16 public LTCFs in Vejle, totaling 863 residents. Four LTCFs, housing 228 residents, are excluded due to non-standardized buildings, shared common areas with kindergartens, and facilities for dementia care. This leaves 12 eligible LTCFs with 635 residents. The allocation phase divides these facilities into three groups: 10 LTCFs with usual care (control) housing 470 residents, 1 LTCF with far-UVC in common rooms housing 76 residents, and 1 LTCF with far-UVC in common and residents’ rooms housing 89 residents. During the follow-up phase, no residents are lost to follow-up in any group, but 3 residents discontinue the intervention in the LTCF with far-UVC in common and residents’ rooms. The intention-to-treat analysis includes all 470 residents from the control group, all 76 residents from the LTCF with far-UVC in common rooms, and 86 residents from the LTCF with far-UVC in common and residents’ rooms.

Navigate back to Figure 1..

## Table 1. Long description

The table presents baseline demographic and clinical characteristics of participants across three groups: Far-UVC in common rooms, Far-UVC in common and residents’ rooms, and usual care. It includes data on age, sex, Charlson comorbidity index score, and vaccine status. The table has 11 rows and 4 columns. The columns are labeled with the group names and the number of participants in each group. The rows are labeled with the characteristics being compared. The age data is presented in categories (<75 years, 75-85 years, >85 years), mean (standard deviation), and median. The sex data is divided into male and female categories. The Charlson comorbidity index score is divided into categories of less than or equal to 1 and greater than or equal to 2. The vaccine status includes pneumococcal, influenza, and COVID-19 vaccines. Notable trends include a higher percentage of older participants in the Far-UVC in common and residents’ rooms group and a higher percentage of females in the Far-UVC in common rooms group.

Navigate back to Table 1..

## Table 2. Long description

The table compares long-term care facilities by group, detailing measures related to building, organization, and resident whereabouts. It includes data on ventilation types, air filtration methods, building age, staff-to-resident ratios, occupant density, cleaning frequency in residents’ homes and common areas, and the whereabouts of residents during different seasons. The table has 20 rows and 4 columns, with headers for characteristics, Far-UVC in common rooms, Far-UVC in common and residents’ rooms, and usual care. Notable trends include the use of coarse and fine filters in facilities with Far-UVC, daily cleaning in common areas for one group, and similar staff-to-resident ratios across all groups.

Navigate back to Table 2..

## Table 3. Long description

The table presents data on infections requiring hospitalization, comparing far-UVC interventions in common rooms and residents’ rooms against usual care. It includes columns for events, incidence rates, unadjusted incidence rate ratios, adjusted incidence rate ratios, and p-values. The table has 6 rows and 8 columns, covering total infections, urinary tract infections, respiratory tract infections, bloodstream infections, and unknown infection focus. Notable trends include significantly lower adjusted incidence rate ratios for total infections and respiratory tract infections in the far-UVC groups compared to usual care.

Navigate back to Table 3..

## Table 4. Long description

The table presents data on antibiotic prescriptions and mortality in long-term care facilities, comparing two intervention groups using far-UVC light in common rooms and common plus residents’ rooms against a usual care group. The table has 7 rows and 10 columns, including headers for outcomes, events, incidence rates (IRs), unadjusted IRRs with 95% confidence intervals, adjusted IRRs with 95% confidence intervals, and P-values. The outcomes measured include total antibiotic prescriptions, urinary tract infections, respiratory tract infections, skin infections, other infections, and mortality. The data shows the number of events and IRs per 10,000 resident days for each group. Notable trends include lower IRRs for total antibiotic prescriptions in the far-UVC groups compared to the usual care group, with adjusted IRRs of 0.52 and 0.83 respectively. The table also highlights specific reductions in urinary tract infection prescriptions in the far-UVC groups. Mortality rates are relatively similar across all groups.

Navigate back to Table 4..

## Table 5. Long description

The table presents results of main and subgroup analyses of hospital-treated infections and antibiotic prescriptions in long-term care facilities among residents aged over 85 years with a Charlson Comorbidity Index (CCI) of 2 or higher. It includes data on infections and antibiotic prescriptions across different treatment groups: far-UVC in common rooms, far-UVC in common and residents’ rooms, and usual care. The table is structured with columns for events, incidence rates (IR), unadjusted IRR with 95% confidence intervals (CI), adjusted IRR with 95% CI, and P-values. Key trends include lower infection rates and antibiotic prescriptions in groups using far-UVC compared to usual care, with notable reductions in specific subgroups.

Navigate back to Table 5..
