# Supplementary material for: The potential effectiveness of far-UVC (222 nm) in preventing infections in long-term care facilities: a six-month nonrandomized controlled phase II trial
Source: Antimicrob Steward Healthc Epidemiol. 2026 Jun 3;6(1):e165. doi: 10.1017/ash.2026.10425 (PMC13237172; doi:10.1017/ash.2026.10425)
Supplement: Kristensen et al. supplementary material [file S2732494X26104252sup001.docx]

| Supplementary 1. Post hoc analysis. Primary and secondary outcomes comparing the intervention group (Far-UVC in common and common and residents' rooms) and the usual care group. Events and incidence rates (IRs) per 10,000 resident days, and unadjusted and adjusted incidence ratios (IRRs) between the pooled intervention groups and the usual care group. | | | | | |
| --- | --- | --- | --- | --- | --- |
| N=635 | Events, IR | Events, IR | Unadjusted IRR (95%CI) | Adjusted^2^ IRR (95%CI) | P-value |
| Outcome | Far-UVC  24740 resident days  (n=165) | Usual care  69585 resident days  (n=470) |  |  |  |
| Infections, all^1^ | 90, 36.4 | 600, 86.2 | 0.4 (0.15–1.2) | 0.4 (0.15–1.1) | 0.086 |
| Urinary tract infections | 27, 10.9 | 172, 24.7 | 0.4 (0.2–1.2) | 0.4 (0.3–0.7) | 0.000 |
| Upper and lower respiratory tract infections | 33, 13.3 | 201, 28.9 | 0.5 (0.2–1.2) | 0.5 (0.2–1.2) | 0.098 |
| Bloodstream infections | 16, 6.5 | 144, 20.7 | 0.3 (0.1–1.2) | 0.3 (0.1–1.1) | 0.067 |
| Unspecified infections | 14, 5.7 | 83, 11.9 | 0.5 (0.1–2.1) | 0.5 (0.1–2.0) | 0.303 |
| Antibiotic prescriptions, all^2^ | 81, 32.7 | 334, 48.0 | 0.68 (0.45–1.02) | 0.68 (0.53–0.87) | 0.002 |
| Antibiotic prescriptions, urinary tract | 37, 15.0 | 157, 22.6 | 0.94 (0.54–1.66) | 0.93 (0.54–1.62) | 0.8 |
| Antibiotic prescriptions, respiratory tract | 37, 15.0 | 129, 18.5 | 1.15 (0.72–1.84) | 1.21 (0.77–1.89) | 0.410 |
| Antibiotic prescriptions, skin | 7, 2.8 | 42, 6.0 | 0.67 (0.23–1.94) | 0.74 (0.26–2.11) | 0.57 |
| Antibiotic prescriptions, other | 0, 0.0 | 6, 0.86 | <5 | <5 | 0.000 |
| Mortality | 23, 9.3 | 78, 11.2 | 0.83 (0.52–1.33) | 0.84 (0.52–1.34) | 0.460 |
| 1: Urinary tract infections, lower respiratory tract infections, bloodstream infections including infections without focus (ICD-10 codes A49, B99), and antibiotics covering unspecified infection sites (J01CA01 and J01GB03 or J01CR05 or J01GB03 and J01DC02) 2: Urinary tract infections, respiratory tract infections, skin infections, and other infections 3: Adjusted for age | | | | | |
